# Assessment of Corrosion Resistance and Hardness of Shot Peened X5CrNi18-10 Steel

**DOI:** 10.3390/ma15249000

**Published:** 2022-12-16

**Authors:** Mariusz Walczak, Mirosław Szala, Wojciech Okuniewski

**Affiliations:** 1 Department of Materials Engineering, Faculty of Mechanical Engineering, Lublin University of Technology, 20-618 Lublin, Poland; 2 Students Research Group of Materials Technology, Department of Materials Engineering, Mechanical Engineering Faculty, Lublin University of Technology, Nadbystrzycka 36, 20-618 Lublin, Poland

**Keywords:** shot peening, X5CrNi18-10, stainless steel, shot peening, corrosion resistance, hardness

## Abstract

Although the application of shot peening facilitates increasing hardness and corrosion resistance of stainless steel, the inappropriate peening parameters result in overestimated hardening and exaggerated surface roughness, which deteriorate the surface morphology and negatively affect the corrosive behavior of treated steel. Therefore it is crucial to select the peening parameters that allow obtaining both high hardness and elevated corrosion resistance. This study aims to determine the effect of X5CrNi18-10 stainless steel samples shot peening on the surface morphology, hardness, and corrosion resistance. Samples were shot peened with a CrNi steel shot, applying 0.3 MPa and 0.4 MPa peening pressures and treatment times of 60 s and 120 s. Roughness analysis and microscopic and SEM-EDS examination were employed to state the effect of peening parameters on the sample’s corrosive behavior in a 3.5% NaCl solution. The most promising shot peening parameters for Vickers hardness and electrochemical corrosion resistance were selected. It is revealed that the surface roughness increase has a detrimental effect on the corrosion behavior. Overall, high corrosion resistance and the high hardness of stainless steel samples were noted for the peening pressure of 0.4 MPa and time treatment of 60 s.

## 1. Introduction

The AISI 300 series austenitic stainless steel have been widely used for critical components operating in marine environments, power plants (light and pressurized water reactors), the medical industry, and the chemical industry due to their excellent mechanical properties and corrosion resistance ability [1]. However, in the case of stainless steel with a specific chemical composition, the properties of the passive layer depend mainly on the surface microstructure [2,3] and the condition of the surface layer [4]. Surface shot peening is an effective treatment to induce localized plastic deformation in the surface layer, which causes grain refinement even to the nanometer scale without changing the chemical composition of the material [4,5]. In addition, this method allows the formation of functionally gradient materials, in which a nanocrystalline surface layer provides suitable surface properties and a coarse-grained core provides ductility [5,6]. Furthermore, shot peening is a relatively simple and inexpensive method of improving the operating characteristics of machine components [7]. Hence, shot peening treatment is successfully used for the surface treatment of various materials, including, but not limited to, titanium [7,8,9], aluminum [10], stainless steel [4,5], magnesium [11,12], etc.

According to the literature data [4,7,8,9], as a result of shot peening processing, shot fragments can penetrate the surface layer (become permanently deposited) and change tribological characteristics [4,9] and corrosion resistance [4,7], and, in the case of components intended for medical use, may show an increase in cytotoxicity [12,13]. Kameyama and Komotori [9] described a model that explains the phenomena occurring in the surface layer of processed metal alloys. This model involves the transfer and mechanical mixing of particle fragments of the shot peening medium in the surface layer. As a result, a locally lamellar microstructure is formed under the influence of the shot peening treatment. The surface layer of the treated material is altered so that the particle striking the material can cause a break in the continuity in the surface material, causing it to break off, as well as shot particles breaking off and remaining on the surface. As a result of the subsequent shot impacts on the surface, the fragments remaining in the surface are more firmly “jammed” into the surface structure of the material, thus creating local strengthening and the lamellar structure characteristic of this type of processing, whose properties are quite different from the core of the material. In the surface layer of austenite-rich steels, an austenitic phase undergoes a strain-induced martensitic transformation [4], and as a consequence, grain refinement can be observed [1,14]. In addition, it has been reported that plastic deformation (which increases surface roughness) can negatively affect corrosion resistance [7]. In contrast, studies [4,14] provide evidence that with certain appropriately selected surface treatment conditions (including size and type of shot, peening pressure, time treatment, etc.), it is possible to achieve refinement of grains in the surface layer that promotes the formation of passive areas and, consequently, it facilitates corrosion resistance. Hence, the extent of changes occurring in the surface layer is a function of the type of stainless steel, type and size of the shot used, frequency of peening, and treatment time [4,7].

Most damage to machine parts (fatigue, abrasive or corrosive wear) originates from defects in the surface layer. Therefore, the condition of the surface layer and the appropriate selection of technological parameters for treatment is a key factor that affects the performance of products operating in corrosive environments [4,5]. Although the application of shot peening facilitates increasing hardness and corrosion resistance of stainless steel, the inappropriate peening parameters result in overestimated hardening, and surface morphology shows too high roughness, which deteriorates the corrosive behavior. Therefore, selecting the peening parameters that enable both high hardness and elevated corrosion resistance is crucial, frequently omitted by the literature and studied separately. This study analyzes the effect of X5CrNi18-10 stainless steel shot peening on the surface morphology, Vickers hardness, and potentiodynamic polarization corrosion testing. The main goal is to determine the most suitable shot peening parameters to obtain both the highest hardness and corrosion resistance of X5CrNi18-10 steel samples.

## 2. Experimental Procedures

### 2.1. Specimens Preparation

**Figure 1:**
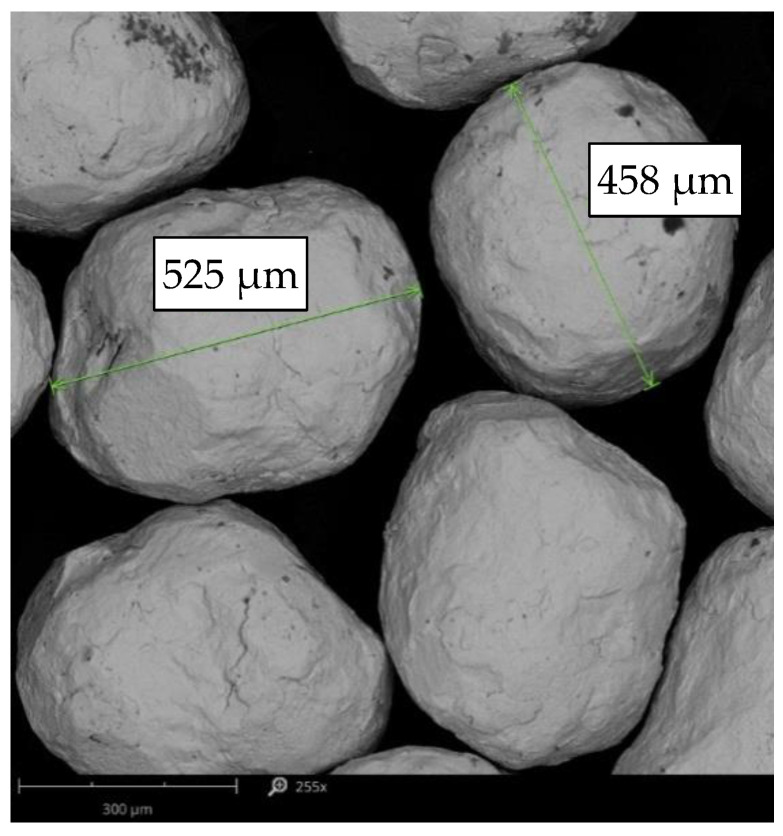
Morphology of stainless steel shot, SEM.

AISI 304 grade austenitic steel (X5CrNi18-10) was used as supplied in the form of a ø25 mm bar. The chemical composition was verified using a Magellan Q8 spark emission spectrometer (Bruker, Germany). The analysis was performed on a detailed Fe130 test channel with 5 analyses (sparks) for each sample. The analyzed steel elements comply with the aforementioned steel grade requirements according to EN 100088-2-2014 (Table 1). Then, the cylinder-shaped samples with a diameter of ø25 mm and a height of 6 mm were cut from the steel rod, which in turn were ground with a Sander/Polisher Saphir 530 metallographic grinder (ATM, Germany) using the following aqueous sandpapers in gradation successively: 300, 500, 800 and 1200. The next step was polishing on polishing cloths with a 3 μm diamond suspension and using 0.04 μm oxide polishing suspension. Subsequently, the external faces of the sample were subjected to a shot peening treatment on the Peenmatic micro 750 S device (IEPCO, Hofstrasse, Switzerland). CrNi stainless steel balls with average sizes of 400 ÷ 900 µm and chemical composition included in Table 2 were used as shot. Average shot sizes oscillate in the range of 400 ÷ 500 µm (Figure 1). In the study, two shot peening pressure were applied: 0.3 MPa and 0.4 MPa at two surface treatment times of 60 s and 120 s. Samples notations are presented in Table 3. The shot peening process was carried out perpendicular to the surface, and the distance of the nozzle from the face of the treated surface was 25 mm. The unpeened stainless steel was used as the reference.

### 2.2. Surface Characterization

The surface roughness after the shot peening treatment was characterized on a Dektak 150 contact profilometer (Veeco Instruments, Plainview, NY, USA). Measurements were taken using a measuring needle with a rounding radius of 2 µm along the sampling line of 5 mm and under a load of 3 mg. The profilometric measurements were performed for each sample at 10 randomly selected places. To assess the degree of development of the surface, the following roughness parameters according to the EN ISO 4287 were estimated: *Ra*—arithmetic average roughness; *Rq*—quadratic mean deviation; *Rv*—maximum profile valley depth of the roughness profile; and *Rp*—maximum profile peak height of the roughness profile. Changes in surface structure morphology were examined using an SEM microscope (Phenom-World, Waltham, MA, USA) at 500× magnifications in topographic mode and employed with an EDS detector used for the chemical composition analysis.

### 2.3. Surface Hardness

The hardness measurements were carried out on the surface of the samples using a Vickers FM-800 microhardness tester with an automatic ARS 900 system (Future-Tech Corp., Kanagawa, Japan). The load used for the hardness test was 1.96 N (HV0.2) with a dwell time of 15 s. For each sample type, twenty indentations were made to obtain statistical accuracy.

### 2.4. Corrosion Test

Corrosion resistance was evaluated quantitatively and qualitatively. Therefore, potentiodynamic polarization testing has succeeded by image analysis of corroded surfaces, which is usually omitted by the literature. Accelerated electrochemical tests determined the corrosion susceptibility of the tested samples in a 3.5% NaCl solution at 22 °C room temperature using the Atlas 0531 corrosion test kit. The corrosion resistance of shot peened and reference steel was investigated in the specified area of 8 mm in diameter, located on the top surfaces of cylinder samples (ø25 mm × 6 mm). Tests were performed in a three-electrode electrochemical vessel, where the counter electrode was a platinum electrode, and the reference electrode was a saturated calomel electrode (SCE). The area of the electrode action was approx. 0.5 cm^2^ (derived from the 8 mm diameter of corrosion testing). The polarization curves were recorded with an automatic potential shift of 1 mV/s in the range from −600 mV to +800 mV. The values of the corrosion currents density *I_corr_* and corrosion potential *E_corr_* were determined from the Tafel plot by analyzing potentiodynamic curves via the AtlasLab software. The corrosion rate (*CR*) was calculated according to ASTM G102 (Standard practice for calculation of corrosion rates and related information from electrochemical measurements) using Equation (1) [15]:

(1) $$CR\;\left(\frac{mm}{year}\right)=3.27\cdot 10\frac{I_{corr}\cdot EW}{\rho }$$

where *I_corr_* is the corrosion current density (μA∙cm^−2^), *ρ* is the material density (g∙cm^−3^), and *EW* is the alloy equivalent weight.

Then, areas of corrosion attack were evaluated using the computer image analysis method. First, corroded areas were subjected to microscopic observations and comparatively analyzed. Then, the percentage of the corroded area was calculated using the Image-Pro software.

## 3. Results and Discussion

### 3.1. Surface Morphology

**Figure 2:**
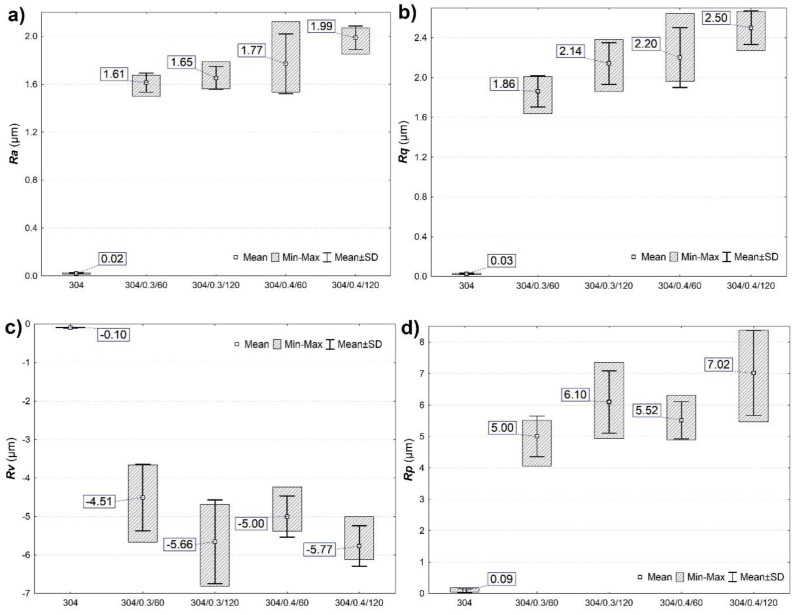
Roughness parameters: (**a**) Ra, (**b**) Rq, (**c**) Rv, and (**d**) Rp of unpeened (marked as 304) and shot peened surfaces.

The roughness of shot peening surfaces was assessed using the most popular and representative roughness parameter *Ra* or *Rq* [8,16,17]. The *Rq* parameter is statistically equal to the standard deviation of the profile ordinates, and a single high elevation and profile cavity affect its value more than the *Ra* value [18]. Figure 2a,b show the surface roughness changes *Ra* and *Rq* of the samples before and after the shot peening process. The surface treatment with steel shot increased the surface roughness both at the pressure of 0.3 MPa and 0.4 MPa. In addition, extending the shot peening time from 60 s to 120 s also increases *Ra* and *Rq*. However, more significant changes in roughness are observed with increasing peening pressure than with increasing machining time. Analyzing the parameters Rp and Rv shows more considerable differences, especially for the sample 304/0.4/60 (Figure 2c,d). In the 304/0.4/60 sample, lower values of peaks and valleys (*Rp* and Rv parameters) are observed compared to the surfaces of 304/0.3/120 and 304/0.4/120. Such surface morphology has a key influence on corrosion performance because surface roughness facilitates corrosive action.

**Figure 3:**
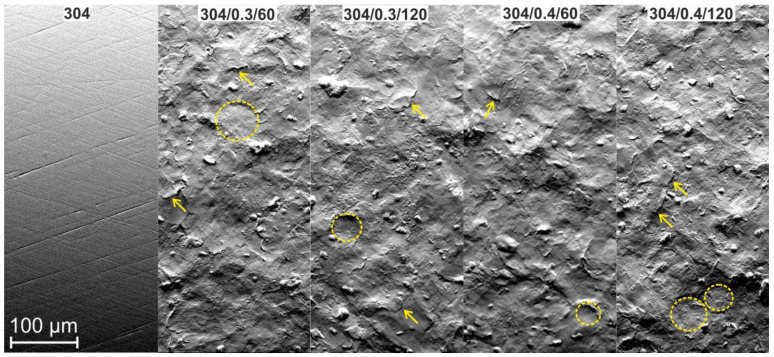
The morphology of specimens surfaces before (sample 304) and after peening with different parameters. Rings indicate spherical CrNi shot bowl imprints; arrows mark overlapping dent, SEM.

SEM microscopic observations revealed that the shot peening processing has seriously modified the initial surface. Surface treatment (Figure 3) causes deformation with visible impact indentations, craters, and spherical CrNi shot bowls imprints. Kameyama et al. [9] indicate that characteristic surface features of shot peened specimens are collision dents covering the specimen completely and increasing the surface roughness.

**Figure 4:**
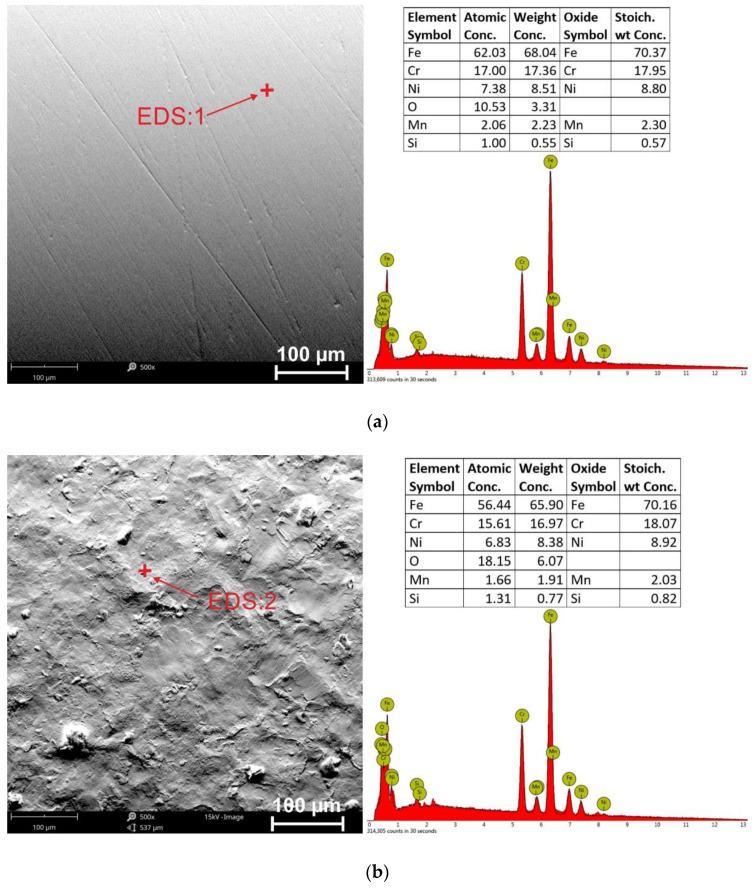
Stainless steel surfaces: (**a**) unpeened (304) and (**b**) shot-peened (sample 304/0.4/60), SEM-EDS.

Increasing the peening pressure to 0.4 MPa intensifies the changes on the surface, leading to an increase in surface development (confirmed by roughness measurements). On the other hand, SEM-EDS chemical composition analysis (Figure 4) revealed higher oxygen values for the surface after the shot peening treatment. The explanation for this is research [14,19,20], which indicates that nanostructured surface layers accelerate the passivation layers formation. The passive layer is denser, more stable, less susceptible to damage by the corrosive environment, and can have a protective effect on the substrate. Gaberšček and Pejovnik [20], on the other hand, showed that passivation film nucleation sites form mainly at twin grain boundaries. Qiao et al. [14] developed a model for the nucleation and formation of passive layers on shot peening surfaces. The microstructure of the untreated sample is an equiaxial structure with a low grain boundary density. The passive film nucleates mainly at these interfaces. The refined grains and high density of grain boundaries are stated in the surface microstructure of samples modified by shot peening. The passive film immediately nucleates on these surfaces and grows uniformly in random directions at the peened surface [1,14].

### 3.2. Microhardness Measurements

**Figure 5:**
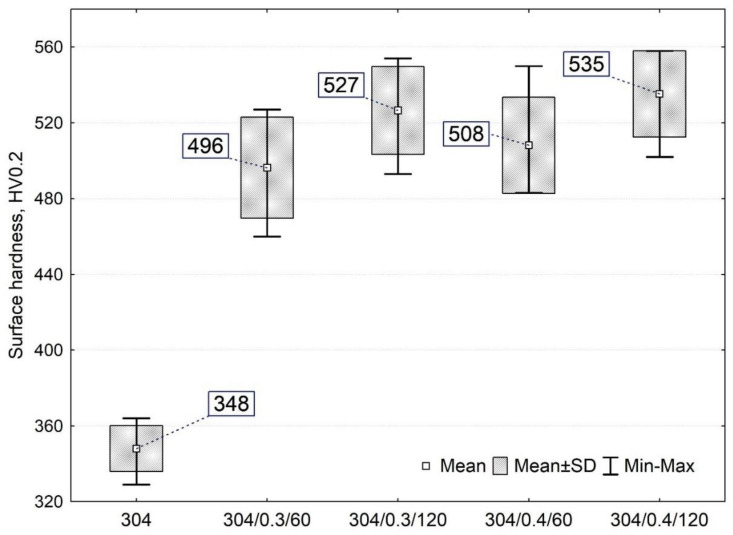
Surface hardness of unpenned (304) and shot peened samples.

Microhardness measurements (Figure 5) showed an increase in average hardness values for all surfaces after shot peening (more than 42%) compared to the untreated specimen. It can be observed that the longer the peening time and the higher the peening pressure, the more significant hardening. Exemplary, at the same peening time, higher hardness is obtained when the peening time increases from 60 s to 120 s (a variation of about 27 ÷ 32 HV0.2) than when the pressure changes from 0.3 MPa to 0.4 MPa (a variation of about 9 ÷ 12 HV0.2). The increase in surface hardness after shot peening can be explained by plastic deformation densifying the dislocations rate, and the remnants of hard shot or, in the case of small diameters, even whole shot particles are pressed in the surface, causing an increase in microhardness [4,9,21]. The model of phenomena occurring in the surface layer due to shot peening is well explained in [9]. The peening medium (shot) hitting the material can cause a break in the continuity in the surface material, causing it to break off and the shot particles to become stuck in a modified surface. Each successive shot hitting the surface deforms the surface layer and forms the characteristic lamellar work-hardened structure. This is a typical microstructure feature of shot-peened metal alloys reported in [22,23,24].

### 3.3. Corrosion Resistance

**Figure 6:**
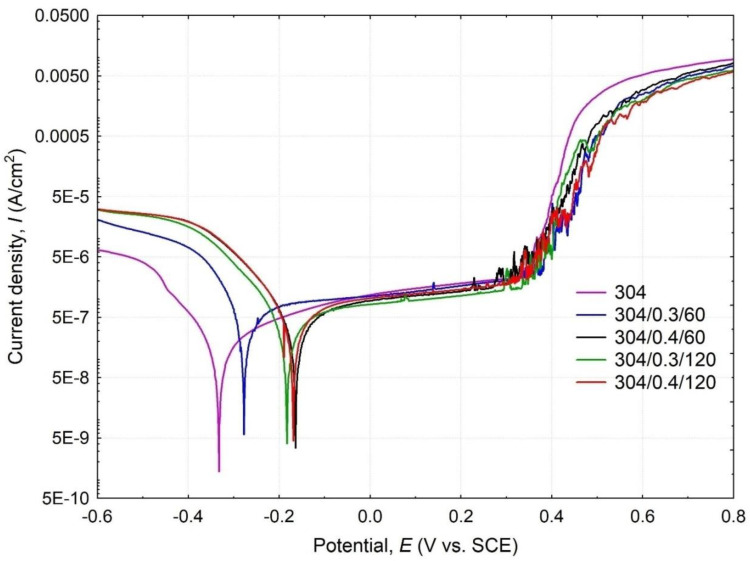
Potentiodynamic polarization curves under open circuit potential condition for reference (304) and shot-peened stainless steel samples in 3.5% NaCl solution.

The results of potentiodynamic polarization tests for surfaces with different shot peening modifications are presented graphically by Tafel curves shown in Figure 6, and characteristic electrochemical parameters are summarized in Table 4. The polarization curve of the 304 sample is comparable to those reported by the literature for AISI 304 steel [25,26,27] but differs from shot-peened samples (Figure 6). Although shot peening effectively increases the hardness of stainless steel, Figure 5, at the same time, the peening process reduces the corrosion resistance shown by standardized corrosion indicators such as polarization resistance *Rp* and corrosion rate *CR* (Table 4). These parameters strongly depend on the corrosion current density (*I_corr_*). At first approximation, the increased corrosion resistance is associated with low *I_corr_*. Therefore, lower overall corrosion resistance is combined with the change in peened surface roughness, increasing the exposed surface area (subjected to corrosive action), which determines the electrochemical results. However, to assess the corrosion resistance of peened samples, the corrosion potential *E_corr_* and the current density *I_corr_* should be considered. Analysis of electrochemical parameters, given in Table 4, indicates that the shot peened 304/0.4/60 specimen seems to have the most favorable corrosion behavior. Hence, this specimen shows two times higher *E_corr_* than the reference sample and relatively low *I_corr_* at a comparable level to the 304 reference sample. In other words, the 304/0.4/60 sample obtained a comparable value of *I_corr_* but significantly more favorable (higher) values of *E_corr_* than those reported for unpeened stainless steel. Furthermore, the decrease in the corrosion resistance of samples after shot peening treatment is generally associated with an increase in corrosion-activated surface area deriving from the changes in surface roughness due to the presence of impact indentations, craters, and spherical imprints. Considering the shot peened samples, the 304/0.4/60 surface show more favorable *Rp* roughness (compared to 304/0.3/120 and 304/0.4/120 surfaces) and *Rv* (compared to 304/0.4/120), limiting the corrosive action. In addition to the roughness effect, corrosion resistance is promoted by the surface layer’s grain refinement and nanocrystal structure. Shot peening usually increases the hardness and results in microstructure refinement, which, according to the literature [1,14], improves corrosion behavior by facilitating the nucleation of the passivation film and improves its compactness.

Studying the shot peening parameters is essential to obtain low surface roughness, high hardness, and elevated corrosion resistance. The literature data [4,28] indicate that the shot peening process increases the surface layer’s hardness and reduces grain size (depending on peening pressure and peening time), which, accompanied by relatively low surface roughness, translates into an increase in corrosion resistance. Furthermore, researchers in many works [1,4,5,7] indicate that shot peening treatment shifts Tafel curves toward positive potential values, which can also be observed in Figure 6. At the same time, in accordance with Balusamy et al. [5], the magnitude of *E_corr_* depends on the appropriate choice of treatment condition (including shot size and peening time, etc.). Therefore, two parallel factors must positively affect corrosion resistance, i.e., low roughness and the surface layer microstructure refinement.

**Figure 7:**
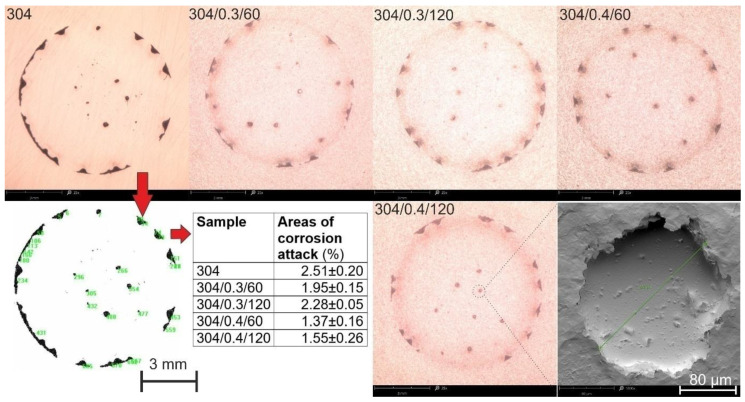
Micrographs of the surface after the potentiodynamic polarization testing in the 3.5% NaCl solution with visible areas of corrosion attack (dark areas).

Microscopic observations and computer image analysis of the surfaces degraded due to corrosion action (Figure 7) revealed different rates of corroded surfaces ranging from 1.37% to 2.51% of the corrosion testing area. The minor corrosion action was reported for 304/0.4/60 surface, which shows the highest corrosion resistance in a 3.5% NaCl solution. Although pitting corrosion is evident for unpeened (304) and shot peened samples, the corroded areas in the ring-like form suggest a typical crevice corrosion behavior characteristic for potentiodynamic tested surfaces [4]. The crevice corrosion appears in the ring-like contact area between the tested surface and sealing ring and is typical for this testing facility design. Moreover, pitting and crevice corrosion of stainless steel in chloride-containing solutions are frequently found together in austenitic stainless steel types [29,30,31]. The tendency for localized corrosion of metals and alloys in aggressive media is usually evaluated through critical potential measurements by using potentiostatic or potentiodynamic electrochemical techniques [32]. The formation of corrosion pits is associated with the occurrence of a breakthrough potential. It is observed in all samples (Figure 6) on the anodic curves in the form of a passivity plateau followed by a sudden increase in current density (passivity breakthrough) and pitting potential (*Ep*) at about Ep ≈ 0.349 ÷ 0.368 V_SCE_ and subsequent development of pitting on the tested surfaces of the samples relating to the breakthrough of the passivated layer. Therefore lower *Ep* has been reported for smoother samples (see Figure 2 and Table 2). It is known that corrosion attack favors initiation at rougher samples [33,34]. Moreover, surface discontinuities or irregularities deriving from the shot peening imprints and dents result in surface morphology and roughness parameter fluctuations (Figure 2 and Figure 3), affecting surface corrosion behavior [35,36,37]. Consequently, the corroded area morphology differs and depends on the initial surface roughness varying from a selection of shot preening parameters.

## 4. Conclusions

This study analyzes the effect of X5CrNi18-10 stainless steel shot peening on the surface morphology, hardness, and corrosion resistance to obtain both high hardness and elevated corrosion resistance. The following conclusions can be drawn based on the experimental results and observations:

Shot peening affects the surface roughness and increases the real surface area affected by the corrosion action. Proper selection of the peening parameters increases hardness and maintains comparable electrochemical stability of peened surfaces. Therefore the 304/0.4/60 sample and unpeened stainless steel (304) show good electrochemical corrosion resistance, represented by standardized corrosion parameters deriving from corrosion current density *I_corr_*.

To thoroughly assess the corrosion resistance of shot peened samples, corrosion potential, *E_corr_*, and current density, *I_corr_*, should be considered. Then, the most favorable corrosion results, i.e., the lowest area of corrosion attack, low current density, and high corrosion potential, are reported for the 304/0.4/60 sample.

SEM-EDS analysis of the surface chemistry of the samples revealed higher oxygen values for the shot peening-treated surfaces, which is due to the formation of a passive film associated with an increase in grain boundary density. In addition, the pitting and crevice corrosion types are identified in the corroded surfaces of unpeened and shot-peened stainless steel surfaces.

The surface roughness and hardness increase with the peening time and increase in the peening pressure.

CrNi steel shot peening causes an increase in the hardness of the machined surface by more than 42% compared to the reference surface. At the same time, higher values of average hardness are obtained when peening time is increased from 60 s to 120 s than when peening pressure is increased from 0.3 MPa to 0.4 MPa.

In summary, the most favorable hardness and corrosion resistance were obtained for the surface treated with CrNi shot at 0.4 MPa and 60 s time (304/0.4/60 sample).

## Figures and Tables

**Table 1:** Comparison measured and nominal chemical composition of AISI 304 (X5CrNi18-10) stainless steel (mas. %).

	C	Mn	Si	P	S	Cr	Ni	N	Fe
EN 10088-2-2014	max 0.030	max 2.00	max 0.75	max 0.045	max 0.015	17.5–19.50	8.00–10.50	max 0.10	bal.
AISI 304 *	0.026	1.545	0.344	0.043	0.001	18.25	8.723	0.064	bal.

* Results of spectrometer analysis.

**Table 2:** Characteristics of shot according to Kuhmichel Abrasiv GmbH.

Shot	Typical Chemical Composition (mas. %)		Grain Size	Grain Shape	Hardness
Stainless steel shot—CrNi	Cr Ni Si Mn C Fe	16.0–20.0 7.0–9.0 1.8–2.2 0.7–1.2 0.05–0.2 bal.	400–900 µm	spherical	235 HV

**Table 3:** Notations of the specimens used in the experiment and shot peening variables.

Specimen Notation	Peening Pressure (MPa)	Peening Time (s)
304	unpeened	
304/0.3/60	0.3	60
304/0.3/120	0.3	120
304/0.4/60	0.4	60
304/0.4/120	0.4	120

**Table 4:** Results of electrochemical corrosion reported for unpeened and shot peened AISI 304 steel.

Sample/Conditions	Corrosion Currents Density, *I_corr_* (µA/cm^2^)	Corrosion Potential, *E_corr_* (V_SCE_)	Pitting Potential, *E_p_* (V_SCE_)	Polarization Resistance, *Rp* (kΩ∙cm^2^)	Corrosion Rate, *CR* (mm/year)
304	0.433	−0.333	0.349	164.185	3.419 × 10^−3^
304/0.3/60	0.555	−0.277	0.353	51.549	4.382 × 10^−3^
304/0.3/120	0.461	−0.183	0.356	77.988	3.641 × 10^−3^
304/0.4/60	0.436	−0.163	0.368	70.805	3.442 × 10^−3^
304/0.4/120	0.455	−0.168	0.362	64.114	3.592 × 10^−3^

## Data Availability

No new data were created or analyzed in this study. Data sharing is not applicable to this article.
